# Femoral varus derotational osteotomy without pelvic osteotomy in nonambulatory children with cerebral palsy: Minimum 5 years follow-up

**DOI:** 10.1097/MD.0000000000028604

**Published:** 2022-01-21

**Authors:** Dai Iwase, Kensuke Fukushima, Yasuaki Kusumoto, Yukie Metoki, Jun Aikawa, Tomonori Kenmoku, Sayoko Minato, Atsushi Matsuo, Masashi Takaso

**Affiliations:** a Department of Orthopaedic Surgery, Kitasato University School of Medicine, Kitasato, Sagamihara City, Kanagawa, Japan; b Department of Physical Therapy, Fukushima Medical University School of Health Sciences, Fukushima City, Fukushima, Japan; c Department of Orthopaedic Surgery, Saga Handicapped Children's Hospital, Kinryu-machi, Saga, Japan.

**Keywords:** cerebral palsy, femoral osteotomy, gross motor function classification system, hip dislocation, hip dysplasia

## Abstract

Whether femoral varus derotational osteotomy (VDRO) alone or a combination of femoral and pelvic osteotomies should be performed for hip dislocation in nonambulatory children with cerebral palsy (CP) remains controversial. Few studies have reported radiographical results after the surgical treatment in nonambulatory children with CP. This study aimed to assess the results and determine predictors indicating progressive hip subluxation and redislocation after VDRO without pelvic osteotomy. We retrospectively analyzed 22 hips in 15 nonambulatory children with CP. All patients underwent VDRO without pelvic osteotomy and were followed up for at least 5 years. The mean follow-up period was 7.3 ± 1.9 years. In radiological assessments, we investigated migration percentage (MP), center-edge angle, neck-shaft angle, teardrop distance, break in Shenton's line (SL), sharp's angle, acetabular ridge angle (ARA), and the change ratio of MP (Change MP). We classified patients with an MP of <40% at final follow-up in the Good group and those with an MP of ≥40% in the Poor group. The Good group included 10 children (14 hips), and the Poor group included 8 children (8 hips). No preoperative differences were found in the means of all the radiographical parameters. However, MP was significantly different between the groups from 1 year postoperatively. ARA showed improvement 5 years after surgery in the Good group. Change MP in the Good group was maintained from immediately after surgery to the final follow-up. Multivariate logistic regression analyses revealed that preoperative break in SL and Change MP immediately after surgery were parameters to predict MP at the final follow-up. In the receiver operating characteristic analysis, the cut-off values were estimated to be 19.2 mm for preoperative SL and 79.0% for Change MP immediately after surgery. Within 7.3 years of follow-up, 63.6% of the patients who underwent VDRO without pelvic osteotomy had good results. Preoperative SL and postoperative Change MP can be considered as predictors of postoperative subluxation and/or dislocation.

## 1 Introduction

**Figure 1:**
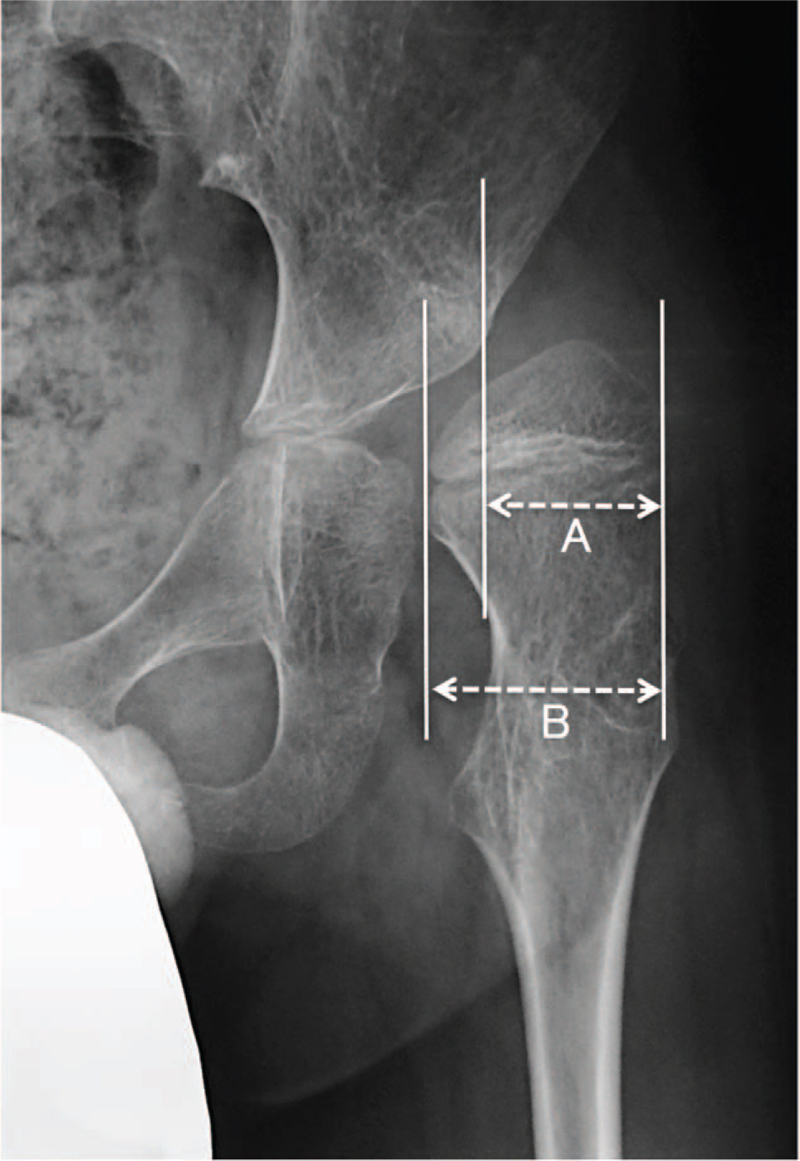
The portion of the femoral head lateral to the Perkins line is measured (A) and expressed as a percentage of the entire width of the femoral head (B). Note that MP = A/B × 100 (%). MP = Migration percentage.

Hip subluxation and/or dislocation is a common problem in children with cerebral palsy (CP). Especially in nonambulatory children with CP, subluxation and/or dislocation frequently occurs because muscle tone is generally hypertonic. Progressive hip subluxation and/or dislocation leads to major complications in these patients, including pain, stiffness, difficulty in positioning, and difficulties with perineal care.^[1]^ Although osteotomy around the hip joint with a soft tissue release (STR) is a standard treatment, it is controversial whether femoral osteotomy alone or a combination of femoral and pelvic osteotomies should be performed. Moreover, few studies have reported clinical results after surgical treatments in nonambulatory children with CP.^[2–4]^ Al-Ghadir et al have reported that although 25% of the patients who received femoral osteotomy alone required revision procedures, none of the patients who received a combined femoral and pelvic osteotomy required further surgery.^[3]^ Consequently, they recommended performing a combination of femoral and pelvic osteotomies rather than femoral osteotomy alone. On the other hand, Terjesen^[4]^ reported no significant difference in the Reimers’ migration percentage (MP) (Fig. 1)^[5]^ at the final follow-up between patients who underwent femoral osteotomy alone and those who underwent the combined surgery. MP represents the percentage of the ossified femoral head lateral to Perkin's line.^[5]^ However, very few studies have investigated radiographic changes during long-term follow-up in patients who underwent femoral osteotomy alone and/or those who underwent a combination of femoral and pelvic osteotomies.

To decrease the surgical invasiveness associated with pelvic osteotomy, we performed STR, open reduction, and femoral varus derotational osteotomy (VDRO) without pelvic osteotomy for hip dislocation in nonambulatory children with CP. In this study, we aimed to assess the mid-term radiographical results and determine predictors indicating progressive hip subluxation and re-dislocation after VDRO without pelvic osteotomy.

## 2 Methods

### 2.1 Patients

This study was approved by the institutional review board of the Kitasato University Hospital (approval number B20–162). The study was performed in accordance with the ethical standards laid down in the 1964 Declaration of Helsinki and its later amendments. The requirement for informed consent was waived because of the retrospective study design.

We retrospectively enrolled 25 children with CP who underwent VDRO at a single institution between January 2004 and December 2014. Of the 25 patients, 21 were nonambulatory, with a Gross Motor Function Classification System (GMFCS) level IV or higher. The exclusion criteria were treatment of hip subluxation or dislocation with VDRO combined with pelvic osteotomy and a follow-up period of less than 5 years. No patients underwent pelvic osteotomy. Six patients could not be followed up for more than 5 years as they relocated elsewhere, among other reasons. Finally, 15 children (22 hips), comprising 10 boys (16 hips), and 5 girls (6 hips), were included in this study. Three children underwent bilateral VDRO; they did not undergo VDRO on the same day. GMFCS at the surgery was level IV in 2 patients and level V in 13 patients.

### 2.2 Surgical procedures

Eight hips underwent STR before VDRO, and 14 hips underwent STR concurrently. Open reduction was performed at the same time as VDRO in all patients. The procedure of STR was the same step in all patients. First, we performed a tenotomy of the biceps femoris, semitendinosus, and semimembranosus from the proximal posterior side in the prone position. Next, we performed fractional lengthening or tenotomy of the adductor longus and myotomy of the gracilis from the proximal medial side, adductor magnus tenotomy from the distal medial side, lengthening or tenotomy of the rectus femoris, fractional lengthening of the iliacus, and psoas major tenotomy from the anterior side in the supine position.^[6]^ Subsequently, open reduction was performed using the anterior approach by cutting the joint capsule and transverse ligament of the acetabulum and by removing the femoral head ligament and acetabular roof soft tissue concurrently. Finally, we performed VDRO in the lateral position. All VDRO procedures were performed with closing wedge resections with a trapezoidal piece of bone and shortening of ≥1 cm. For fixation, we used the Angle blade plates (DePuy Synthes, Japan). The target correction angles were 100° to 110° of the neck-shaft angle (NSA) and 10° of anteversion immediately after surgery. Postoperatively, a hip spica cast was applied and maintained for 6 weeks, followed by the application of a hip abduction brace for 4 months. After the hip spica cast was removed, the patients underwent physical therapy to prevent postoperative joint contracture. In particular, we focused on improving hip flexion, abduction and external rotation.

### 2.3 Radiological assessments

Anteroposterior radiographs of the pelvis and hip joints were taken preoperatively and postoperatively with the patients in the supine position.

**Figure 2:**
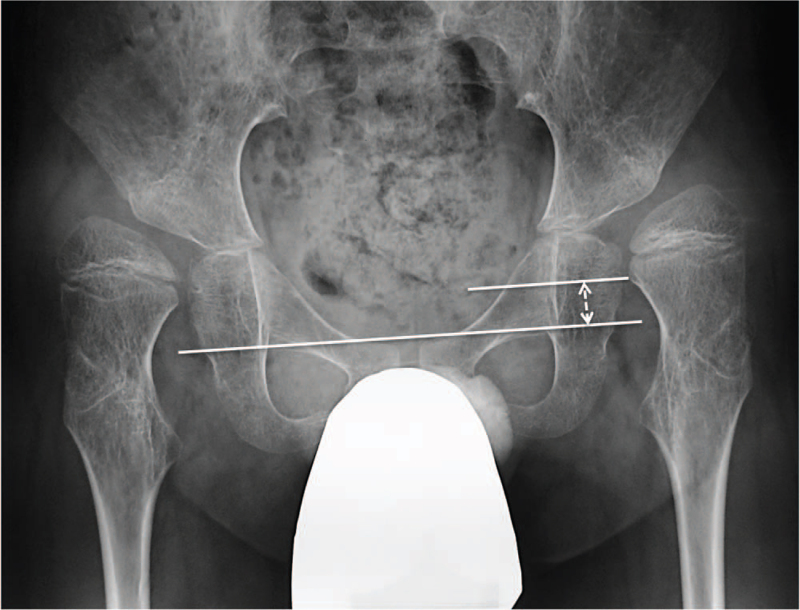
Break in SL. SL is an imaginary curved line drawn along the inferior border of the superior pubic ramus along the inferomedial border of the neck of the femur. Break in SL is defined as plus (mm) when the inferomedial border of the neck of the femur moves above the inferior border of the superior pubic ramus. The white arrow between the two lines indicates a break in SL with a plus value. SL = Shenton's line.

The following parameters were measured: MP, center-edge angle (CEA), NSA, teardrop distance (TDD),^[7]^ a break in Shenton's line (SL), sharp's angle (SA), acetabular ridge angle (ARA),^[8]^ and the change in the MP ratio (Change MP). SL is an imaginary curved line drawn along the inferior border of the superior pubic ramus (superior border of the obturator foramen) along the inferomedial border of the neck of the femur (Fig. 2).^[9]^ Movement of the inferomedial border of the neck of the femur above the inferior border of the superior pubic ramus was defined as plus (mm). Change MP was the ratio of the change in the postoperative MP value relative to the preoperative MP value. All measurements were performed using a picture archiving and communication system (Yokogawa Medical Solutions Corporation, Tokyo, Japan). All radiological assessments were performed independently by two observers blinded to the treatment outcomes, and the averaged values were recorded.

MP, CEA, NSA, TDD, SL were measured preoperatively; immediately after surgery; and 1, 3, and 5 years postoperatively and at the final follow-up. In addition, SA and ARA were measured preoperatively and 1, 3, and 5 years postoperatively and at the final follow-up.

We defined the patients with an MP of <40% at the time of final follow-up as the Good group and those with an MP of ≥40% as the Poor group.

### 2.4 Statistical analyses

Results are expressed as mean and standard deviation of the mean unless otherwise indicated. The normality of all data was initially confirmed using a histogram. The participants’ characteristics (sex, timing of STR, age at surgery, and follow-up period) were analyzed using a Student's *t* test and Pearson chi-square test.

To investigate the characteristics of the parameters preoperatively and postoperatively, each parameter was assessed using two-way analysis of variance, simple main effects, and a multiple comparison test using the Bonferroni method, with the groups as the factor without correspondence and before and after surgery as the factor with correspondence. Next, we investigated factors contributing to the influential factors behind the study's clinical result with as few variables as possible. Variables with a *P*-value of <.2 in a two-group comparison up to 1 year postoperatively were used as independent variables, and the Good and Poor MP groups were used as dependent variables. These associations were assessed using multivariate logistic regression analyses with stepwise forward selection (likelihood ratio). A receiver operating characteristic (ROC) analysis was performed to determine the diagnostic cut-off values for the improvement of hip dislocation in selected parameters using multivariate logistic regression analyses. Statistical analyses were performed using IBM SPSS Statistics for Windows, ver. 27 (IBM Corp, Armonk, NY). A *P-*value of ≤.05 was considered to imply statistical significance.

## 3 Results

The patients’ characteristics are presented in Table 1. Their mean age at the time of surgery was 8.4 ± 2.3 years. The mean follow-up period was 7.3 ± 1.9 years.

**Table 1:** Characteristics of the participants.

	Good group (n = 14 hips)	Poor group (n = 8 hips)	*P*
Sex male, female; n (hip)	11, 3	5, 3	.42
^∗^Timing of STR (pre, same); n (hip)	5, 9	3, 5	.93
Age at surgery, years	7.7 (±1.9)	9.1 (±4.9)	.43
Follow up period, years	8.3 (±2.0)	6.7 (±1.2)	.06

According to the radiological assessments of the MP value, 10 children (14 hips) were assigned to the Good group and 8 (8 hips) were assigned to the Poor group. Three children each in the Good and Poor groups (six hips) underwent bilateral VDRO. The participants’ characteristics were not significantly different (*P* > .05) between the groups (Table 1).

No preoperative differences were observed in the means of all radiographic parameters. A main effect was found for all parameters, except SA, and no interaction was found for all parameters in the statistical analysis. SA in the Good and Poor groups and ARA in the Poor group were not significantly different (*P* > .05) between the two groups. However, MP and Change MP at 1, 3, and 5 years postoperatively and at the final follow-up; CEA at 3 and 5 years postoperatively and at the final follow-up; TDD 5 years postoperatively; and SL and SA 5 years postoperatively and at the final follow-up showed significant differences between the two groups (*P* < .05; Table 2). Parameters with *P*-values <.2 for each parameter up to 1 year postoperatively were preoperative SL and SA at 1 year postoperatively.

**Table 2:** Table postoperative course of each parameter between the Good and Poor groups.

		Pre	Po	Po 1y	Po 3y	Po 5y	Final
MP	Good	85.7 ± 21.9 [100.0 (61.5, 100.0)]	17.6 ± 20.9^b^ [12.9 (0.0, 24.6)]	20.8 ± 17.4^b^ [15.0 (3.8, 34.8)]	28.5 ± 11.0^b^ [28.5 (17.8, 38.4)]	29.4 ± 8.4^b^ [30.5 (22.9, 34.9)]	28.7 ± 7.8^b^ [30.7 (21.9, 35.0)]
	Poor	98.3 ± 5.0 [100.0 (100.0, 100.0)]	27.8 ± 13.2^b^ [30.0 (17.0, 42.4)]	43.4 ± 15.9^a^^,^^b^ [40.9 (29.3, 61.5)]	52.5 ± 16.8^a^^,^^b^^,^^c^ [51.7 (40.5, 61.2)]	54.8 ± 15.3^a^^,^^b^^,^^c^ [54.5 (44.9, 58.6)]	54.5 ± 20.1^a^^,^^b^^,^^c^ [47.0 (42.3, 62.0)]
CEA	Good	–52.7 ± 38.0 [–56.5 (–92.5, –14.0)]	8.3 ± 10.7^b^ [9.0 (5.9, 17.0)]	9.1 ± 8.4^b^ [11.8 (5.5, 15.6)]	11.6 ± 7.2^b^ [10.7 (8.0, 13.8)]	13.9 ± 8.4^b^ [12.5 (8.0. 18.3)]	16.1 ± 8.4^b^ [12.3 (10.0, 25.5)]
	Poor	–56.3 ± 32.9 [–47.0 (–55.5, –35.0)]	4.9 ± 13.5^b^ [7.5 (–1.4, 10.8)]	0.8 ± 16.7^b^ [4.0 (–16.6, 11.0)]	–3.1 ± 17.2^a^^,^^b^ [–0.5 (–11.6, 9.4)]	–3.9 ± 16.6^a^^,^^b^ [–2.5 (–8.3, 9.4)]	–5.6 ± 21.5^a^^,^^b^ [2.5 (–11.8, 10.1)]
NSA	Good	163.1 ± 10.8 [163.5 (150.8, 175.0)]	116.8 ± 8.6^b^ [114.5 (110.0, 124.3)]	122.1 ± 11.9^b^ [121.0 (112.3, 128.5)]	127.9 ± 11.9^b^^,^^c^ [127.5 (116.5, 138.0)]	129.2 ± 12.9^b^^,^^c^ [128.0 (118.8, 141.0)]	128.7 ± 11.4^b^^,^^c^ [125.0 (122.3, 141.0)]
	Poor	160.9 ± 11.9 [163.0 (158.3, 172.3)]	116.5 ± 12.0^b^ [113.5 (105.3, 125.0)]	124.6 ± 10.2^b^ [125.5 (116.8, 131.5)]	129.1 ± 12.1^b^^,^^c^ [132.0 (116.8, 137.8)]	128.1 ± 14.1^b^ [127.5 (118.8, 143.0)]	128.3 ± 14.6^b^ [129.0 (118.0, 143.2)]
TDD	Good	20.1 ± 6.2 [23.2 (15.7, 25.9)]	8.7 ± 2.5^b^ [8.3 (6.5, 10.0)]	8.5 ± 2.7^b^ [7.0 (5.8, 11.2)]	7.3 ± 2.3^b^ [7.1 (4.8, 9.1)]	7.0 ± 2.8^b^ [6.4 (4.5, 9.2)]	6.8 ± 3.2^b^ [7.3 (3.0, 8.7)]
	Poor	20.9 ± 5.2 [18.5 (15.6, 23.0)]	8.4 ± 2.4^b^ [9.5 (6.4, 10.8)]	9.2 ± 4.4^b^ [9.7 (6.0, 14.0)]	9.4 ± 5.0^b^ [8.5 (5.9, 14.4)]	10.9 ± 5.4^a^^,^^b^ [9.6 (7.6, 15.2)]	10.7 ± 5.8^b^ [9.4 (6.8, 17.2)]
SL	Good	16.0 ± 7.0 [21.1 (13.1, 23.6)]	–1.8 ± 2.9^b^ [–2.5 (–5.2, 0.0)]	–0.7 ± 4.1^b^ [0.0 (–4.1, 0.5)]	–1.7 ± 6.0^b^ [–1.5 (–5.3, 2.0)]	–2.0 ± 5.4^b^ [0.0 (–8.2, 3.5)]	–1.1 ± 6.5^b^ [0.0 (–8.2, 3.5)]
	Poor	23.9 ± 12.2 [16.5 (15.5, 18.8)]	–3.6 ± 2.3^b^ [–2.6 (–4.2, –0.5)]	0.9 ± 3.7^b^ [2.0 (0.0, 4.9)]	4.7 ± 8.9^b^ [5.0 (3.0, 9.5)]	5.4 ± 10.3^a^^,^^b^ [5.5 (1.1, 11.2)]	5.1 ± 11.7^a^^,^^b^ [4.1 (1.0, 10.5)]
SA	Good	49.1 ± 6.2 [48.7 (44.9, 52.1)]	–	49.5 ± 5.2 [47.8 (46.3, 51.7)]	49.3 ± 4.3 [49.0 (45.7, 53.0)]	48.9 ± 4.1 [48.7 (45.5, 51.4)]	48.0 ± 4.5 [48.0 (44.5, 50.1)]
	Poor	51.2 ± 4.3 [52.5 (49.6, 57.1)]	–	51.9 ± 4.3 [53.7 (48.3, 56.3)]	53.0 ± 4.0 [52.6 (52.1, 55.6)]	53.6 ± 3.5^a^ [53.7 (51.5, 54.8)]	52.0 ± 3.4^a^ [51.5 (49.5, 55.4)]
ARA	Good	–17.5 ± 9.5 [–14.4 (–20.3, –12.0)]	–	–14.7 ± 10.3 [–13.0 (–17.8, –7.5)]	–11.2 ± 9.6 [–11.9 (–14.9, –8.0)]	–5.2 ± 10.6^b^^,^^d^^,^^e^ [–6.2 (–12.8, 3.9)]	–3.4 ± 10.0^b^^,^^d^^,^^e^ [–4.2 (–12.3, 7.3)]
	Poor	–18.2 ± 9.9 [–16.5 (–33.5, –9.2)]	–	–15.2 ± 8.2 [–15.5 (–27.3, –8.1)]	–12.9 ± 9.4 [–16.3 (–21.3, –7.6)]	–13.9 ± 8.9 [–17.5 (–19.7, –10.4)]	–12.3 ± 10.8 [–14.9 (–18.6, –0.5)]
Change MP	Good	–	79.2 ± 23.3 [84.6 (68.4, 100.0)]	76.3 ± 16.9 [70.7 (65.3, 92.5)]	66.9 ± 8.3 [66.3 (61.7, 71.1)]	63.9 ± 12.1 [65.2 (55.0, 75.5)]	64.3 ± 12.7 [66.4 (55.8, 71.0)]
	Poor	–	71.8 ± 13.0 [70.0 (57.6, 82.2)]	56.0 ± 15.3^a^ [59.1 (38.6, 69.4)]	46.6 ± 16.4^a^^,^^c^ [48.2 (38.8, 57.8)]	44.2 ± 15.0^a^^,^^c^ [44.1 (41.4, 53.9)]	44.7 ± 19.6^a^^,^^c^ [51.0 (38.0, 57.0)]

**Figure 3:**
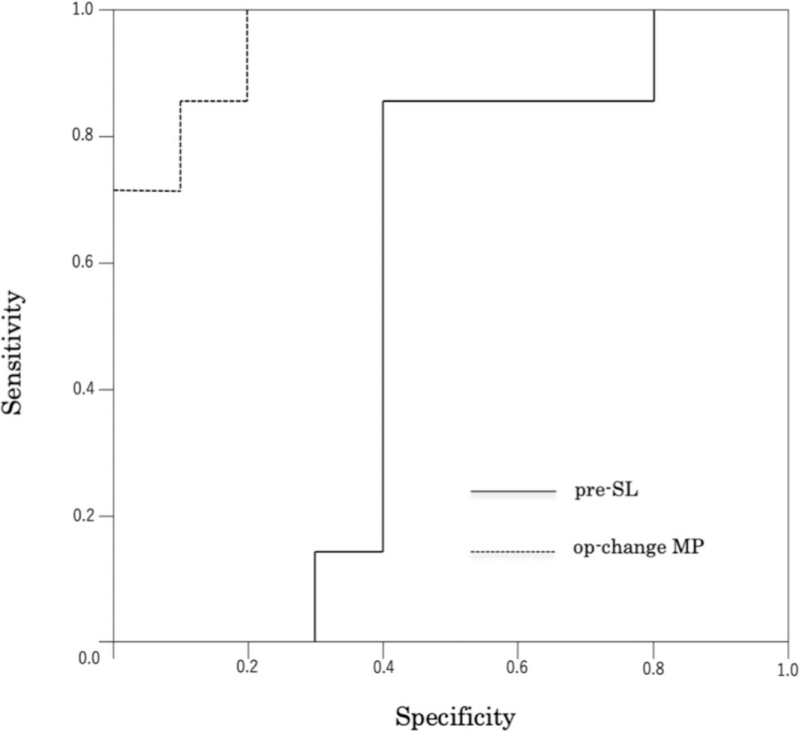
Multivariate logistic regression analyses. The cut-off values were 19.2 mm for pre-SL (AUC ; 0.557, sensitivity; 0.857, specificity; 0.600) and 79.0% for op-change MP (AUC; 0.957, sensitivity; 0.857, specificity; 0.900). pre-SL; preoperative SL, op-change MP; Change MP immediately after surgery. AUC = area under the curve.

Based on the results of the multivariate logistic regression analyses, preoperative SL and Change MP immediately after surgery were selected as parameters to predict the MP value at the final follow-up. In the ROC analysis, the cut-off values were estimated as 19.2 mm for preoperative SL (area under the curve [AUC], 0.557; sensitivity, 0.857; specificity, 0.600) and 79.0% for Change MP immediately after surgery (AUC, 0.957; sensitivity, 0.857; specificity, 0.900; Fig. 3).

## 4 Discussion

The treatment of hip dislocation in children with CP is intended to maintain a well-positioned, supple, and painless hip joint. Particularly for nonambulatory children, improving positioning efforts for perineal care is vital. Shore et al^[10]^ and Terjesen^[11]^ suggested that preventive surgery with STR usually provides a satisfactory outcome in ambulatory children with moderate degrees of hip displacement. However, because the outcome deteriorates in nonambulatory children (GMFCS levels of IV and V) with a more pronounced hip displacement, nonambulatory children might often require osseous reconstructive hip surgery.^[10,11]^ Only a few studies on nonambulatory children^[2–4]^ have reported radiographic results with long-term follow-up data. Our study evaluated radiographic results in nonambulatory children with CP with an average of 7.3 years of follow-up.

In a systematic review of bone reconstruction for CP with GMFCS levels of III to V (36 studies), all patients received STR. However, there are various STR methods. In general, STR includes the release of the adductor muscle with or without releasing iliopsoas muscle. Six studies used routine iliopsoas tenotomy with or without adductor release. Only four studies (4/36; 11%) included an added hamstring release.^[12]^ The vector of the resulting force of the hip joint in patients with CP is directed laterally, superiorly, and posteriorly. In severe CP, the adductors, iliopsoas, and medial hamstrings have a powerful hip dislocation effect.^[13]^ Moreover, the hamstring was proximally dissected to facilitate femoral head pulling.^[6]^ Therefore, we treated the biceps femoris, semitendinosus, semimembranosus, adductor longus and gracilis, adductor magnus, rectus femoris, iliacus, and psoas major muscles. In addition, the indication for open reduction remains controversial. Some researchers^[14,15]^ recommend open reduction, while others do not.^[4,16]^ We performed open reduction in all patients because we wanted to easily pull down the femoral head along with a proximal hamstring separation. Despite such an intensive STR and open reduction, eight hips showed poor outcomes. These results suggest that the effectiveness of soft tissue treatment might be limited compared with that of bony corrective surgery.

There is a clear consensus on performing VDRO to treat hip dislocation in nonambulatory children with CP. However, the indication and necessity to add pelvic osteotomy remain controversial. Shore et al^[17]^ suggested that nonambulatory children with GMFCS levels of IV and V require revision hip surgery more often than ambulatory children with GMFCS levels of I, II, and III after performing VDRO alone. Conversely, Terjesen^[4]^ reported that the outcome after combined osteotomies was not significant compared with that after VDRO alone. In our results, 36% of the patients were categorized in the Poor group. The percentage of poor results was similar to findings of previous reports of nonambulatory patients with CP (25–46%).^[3,4,17]^ From the results, we considered that some nonambulatory children might be required pelvic osteotomy.

We also attempted to identify the radiological parameters to determine the necessity of pelvic osteotomy and to predict good clinical results. None of the preoperative parameters showed significant differences between the Good and Poor groups. All parameters of both groups immediately after surgery showed significant improvement. MP values were significantly different between the groups from 1 year postoperatively. While preoperative MP has been described as a predictor for poor outcomes in some studies,^[18,19]^ this was not applicable in our study. ARA showed improvement 5 years after surgery in the Good group. Acetabular remodeling for spastic CP has shown limited potential in contrast to that for routine developmental dysplasia of the hip.^[3]^ Hoffer et al^[20]^ described that hips with preoperative dislocation do not display acetabular remodeling after performing VDRO alone. In this study, ARA had a large standard deviation; therefore, it was unclear whether ARA had improved. Change MP in the Good group remained preserved immediately after surgery to final follow-up, and Change MP immediately after surgery was selected by multivariate logistic regression analyses. Park et al suggested that postoperative MPs > 5.1% were inflection points for hip redislocation after VDRO without pelvic osteotomy in nonambulatory children with CP. Their finding indicated that severely subluxated or dislocated hips and hips in which the femoral head is successfully reduced by VDRO, but is still contained within the dysplastic acetabulum, may benefit from the additional pelvic osteotomy.^[21]^ Our results suggested that if Change MP immediately after VDRO was ≥79%, VDRO alone might maintain the repositioning of the hip until the final follow-up. Because Change MP could be confirmed intraoperatively, a Change MP of ≤79% after VDRO might be an intraoperative predictor that should add a pelvic osteotomy. In our study, preoperative break in SL was also selected using multivariate logistic regression analyses. Chang et al^[18]^ evaluated breaks in the SL. They reported 16% (29/179) hips with breaks in the SL at final follow-up. It might be challenging to maintain a reposition without large varus correction and/or shortening osteotomy in patients’ hips with large breaks in the SL. We considered that additional pelvic osteotomy might be required for hips if the preoperative break in SL value exceeds 19.2 mm.

There are some limitations of the study. First, this study was a single-center retrospective series assessing a small number of patients, thus limiting the external validity of the findings of the two-way analysis of variance and ROC analysis. Second, clinical outcomes were not reported. Since children have limited ability to express the presence and intensity of pain, it was difficult to assess pain in patients with severe CP clinically. Finally, we performed radiographic assessments with only plain radiographs because of difficulty in resting and radiation exposure. Considering the age when surgery was performed and the skeletal immaturity during childhood, further assessment methods might be considered. Regardless of these limitations, we believe that our study enables us to draw meaningful conclusions.

In summary, within an average of 7.3 years follow-up, 63.6% (14/22) hips in the nonambulatory children with CP had good results after VDRO without pelvic osteotomy. Our findings suggest that pelvic osteotomy might be required in patients with a preoperative break in SL of ≥19.2 mm and/or a Change MP value of ≤79% immediately after VDRO.

## Acknowledgments

We would like to thank Editage [http://www.editage.com] for editing and reviewing this manuscript for English language.

## Author contributions

**Conceptualization:** Dai Iwase, Kensuke Fukushima, Yukie Metoki, Jun Aikawa, Atsushi Matsuo.

**Data curation:** Dai Iwase, Yukie Metoki, Jun Aikawa, Tomonori Kenmoku, Sayoko Minato, Atsushi Matsuo.

**Formal analysis:** Yasuaki Kusumoto.

**Funding acquisition:** Dai Iwase, Jun Aikawa, Tomonori Kenmoku, Masashi Takaso.

**Investigation:** Dai Iwase, Kensuke Fukushima, Yukie Metoki, Jun Aikawa, Tomonori Kenmoku, Sayoko Minato.

**Methodology:** Dai Iwase, Kensuke Fukushima, Jun Aikawa, Tomonori Kenmoku, Atsushi Matsuo.

**Project administration:** Dai Iwase, Kensuke Fukushima, Jun Aikawa, Tomonori Kenmoku, Atsushi Matsuo.

**Resources:** Jun Aikawa, Tomonori Kenmoku, Masashi Takaso.

**Software:** Yasuaki Kusumoto.

**Supervision:** Masashi Takaso.

**Validation:** Yasuaki Kusumoto.

**Visualization:** Dai Iwase, Yukie Metoki, Jun Aikawa.

**Writing – original draft:** Dai Iwase, Kensuke Fukushima.

**Writing – review & editing:** Dai Iwase, Kensuke Fukushima, Yasuaki Kusumoto, Yukie Metoki, Jun Aikawa, Tomonori Kenmoku, Sayoko Minato, Atsushi Matsuo, Masashi Takaso.
